# Impact of the Combination of Remimazolam and Volatile Anesthetics on Circulatory Dynamics in Free-Flap Reconstruction Surgery for Oral Cancer: A Retrospective Comparison With Desflurane and Sevoflurane

**DOI:** 10.7759/cureus.96877

**Published:** 2025-11-14

**Authors:** Yoko Sasaki, Hideyuki Nakagawa, Akira Kitamura

**Affiliations:** 1 Department of Anesthesiology, Saitama Medical University International Medical Center, Saitama, JPN; 2 Department of Dentistry, Saitama Rehabilitation Center, Saitama, JPN

**Keywords:** combination, desflurane, free-flap reconstruction, remimazolam, sevoflurane

## Abstract

Background

Patients who undergo free-flap reconstruction surgery for oral cancer are often elderly and require prolonged surgical procedures, making intraoperative circulatory management critical. We investigated the suitability of combining remimazolam besylate (remimazolam), which has minimal circulatory depressant effects, with inhalational anesthetics for maintenance anesthesia in comparison to maintenance using inhalational anesthetics alone.

Methodology

A retrospective analysis was conducted of patients who underwent oral tumor resection, neck dissection, and free-flap reconstruction surgery at a single institution between October 1, 2020, and April 30, 2022. Patients were divided into the following three groups according to anesthetic use: remimazolam and desflurane combination (Des+R), desflurane monotherapy (Des), and sevoflurane monotherapy (Sevo). Vital signs, vasopressor use, blood loss, and presence or absence of flap complications requiring reoperation were compared.

Results

Data from 47 patients (Des+R, n = 14; Des, n = 18; Sevo, n = 15) were analyzed. The mean arterial pressure in Des+R was significantly higher than in Des before reconstruction and higher than in both Des and Sevo during vascular anastomosis. Continuous vasopressor use was significantly less frequent in the Des+R group (pre-reconstruction: 21% vs. 77% and 60%, p = 0.006; vascular anastomosis: 28% vs. 72% and 66%, p = 0.04). Ephedrine use was greater in the Des group (p = 0.02), while phenylephrine use did not differ (p = 0.8). Blood loss was greater in Des+R than in Sevo (p = 0.04). Reoperations due to flap complications (thrombosis, hematoma, necrosis) were performed in Des+R (two patients, three times), Des (three patients, three times), and Sevo (three patients, five times).

Conclusions

In free-flap reconstruction surgery for oral cancer, the combination of desflurane and remimazolam anesthesia maintained higher blood pressure and stable hemodynamics while reducing vasopressor requirements. Given the small sample size and retrospective design, no definitive conclusions about superiority can be drawn. Well-designed prospective studies with standardized vasopressor protocols and assessment of postoperative flap complications are needed to further evaluate the suitability of this combination.

## Introduction

Patients who undergo free-flap reconstruction surgery for oral cancer are often elderly, and the surgical procedure is often lengthy. Additionally, many patients have underlying medical conditions, making it critical to conduct appropriate circulatory management during surgery. Avoiding intraoperative hypotension has been reported to potentially reduce the incidence of postoperative cardiovascular complications and delirium [1,2]. On the other hand, maintaining blood pressure at elevated levels may increase the bleeding volume. In free-flap reconstruction surgery, blood pressure management to maintain flap perfusion and appropriate fluid management are needed. Furthermore, there is ongoing debate regarding the vasodilatory and circulatory depressant effects of anesthetic agents, and the optimal anesthetic technique has not yet been established [3].

General anesthesia management involves the use of inhalation anesthetics or intravenous anesthetics, each with its own advantages and disadvantages. Compared with intravenous anesthetics, inhaled anesthetics have a slower onset but are rapidly exhaled from the lungs upon cessation of administration, allowing for easier adjustment of anesthetic depth. Intravenous anesthetics have a rapid onset but slow metabolism and excretion, resulting in poor control of anesthetic depth. Total intravenous anesthesia using short-acting propofol has become widely adopted, but its strong circulatory depression is a drawback.

Recently, remimazolam besylate (remimazolam), a short-acting intravenous anesthetic with minimal circulatory depression, was introduced [4], and it has been reported to cause fewer episodes of hypotension than propofol during anesthesia induction [5-7]. However, remimazolam alone may cause significant fluctuations in blood pressure and heart rate depending on the degree of surgical trauma [8].

In this retrospective study, we hypothesized that the combination of remimazolam and desflurane would be advantageous for stabilizing hemodynamics in free-flap reconstruction surgery for patients with oral cancer. The purpose of this study was to elucidate the hemodynamic effects of the combined use of remimazolam and desflurane. Accordingly, we compared intraoperative hemodynamics between commonly used inhalational anesthetics (desflurane or sevoflurane) and the combination of remimazolam with desflurane in patients undergoing free-flap reconstruction surgery for oral cancer.

## Materials and methods

This study was conducted after obtaining the approval of the Institutional Review Board of Saitama Medical University International Medical Center (approval number: 2022-012).

Inclusion and exclusion criteria

A retrospective analysis was performed of patients who underwent oral tumor resection, neck dissection, and free-flap reconstruction surgery at our institution between October 1, 2020, and April 30, 2022. Patients in whom propofol was used as a maintenance anesthetic were excluded from the study because only one case of complete intravenous anesthesia with propofol was performed during the period. Patients on dialysis were also excluded from the study because their infusion volume and circulatory control were different.

Data collection and calculation

Patient information was collected from electronic medical records, and surgical information, anesthetic management methods, and vital sign data were collected from anesthetic records. Patient information included age, sex, underlying disease, pre-induction noninvasive mean arterial pressure and heart rate, preoperative serum hemoglobin, and whether reoperation was required within one week after surgery due to flap complications. Surgical information, anesthetic management methods, and vital sign data included mean arterial pressure, heart rate, bispectral index (BIS) values, fluid infusion volume, blood loss, urine output, anesthetic duration, surgical duration, type of anesthetic agent, inhaled anesthetic agent concentrations (expiration), type of vasopressor, dosage, and administration method. These data were compared across the following three groups according to the type of anesthetic agent used: the desflurane plus remimazolam group (Des+R group), the desflurane alone group (Des group), and the sevoflurane alone group (Sevo group). These anesthetics are the standard anesthetics used at our facility.

Vital sign data were calculated as the average of data recorded every minute from the automated anesthesia recorder. The BIS value and end-tidal anesthetic gas concentration were calculated as the average over the entire period from the start to the end of surgery. The mean arterial pressure and heart rate were calculated separately for the “pre-reconstruction” period (from the start of surgery to cervical dissection and tumor resection) and for the “vascular anastomosis” period (from the start to the completion of vascular anastomosis).

Statistical analysis

Statistical analysis was performed using R (ver. 4.2.1), and p-values <0.05 were considered statistically significant. The three anesthesia methods were tested for differences in each parameter. When significant differences were found, multiple comparison tests were performed. For categorical variables, differences between groups were evaluated using Fisher’s exact probability test. For continuous variables, normality was confirmed using the Shapiro-Wilk test, and homogeneity of variance was confirmed using Bartlett’s test. If normality and homogeneity of variance were confirmed, group comparisons were performed using analysis of variance (ANOVA) and the Tukey-Kramer method. If homogeneity of variance was not confirmed, group comparisons were performed using the Games-Howell method. For non-normally distributed data, the Kruskal-Wallis test and Steel-Dwass method were used.

Randomization was not performed because the choice of anesthetic was optional. Additionally, no matching was performed because hemodialysis patients with clearly different circulatory control were excluded. Some patients could not be fitted with a BIS monitor due to the nature of their surgery, and because there was missing data in the BIS, we did not perform a comparison between groups.

## Results

Patient demographics

**Figure 1:**
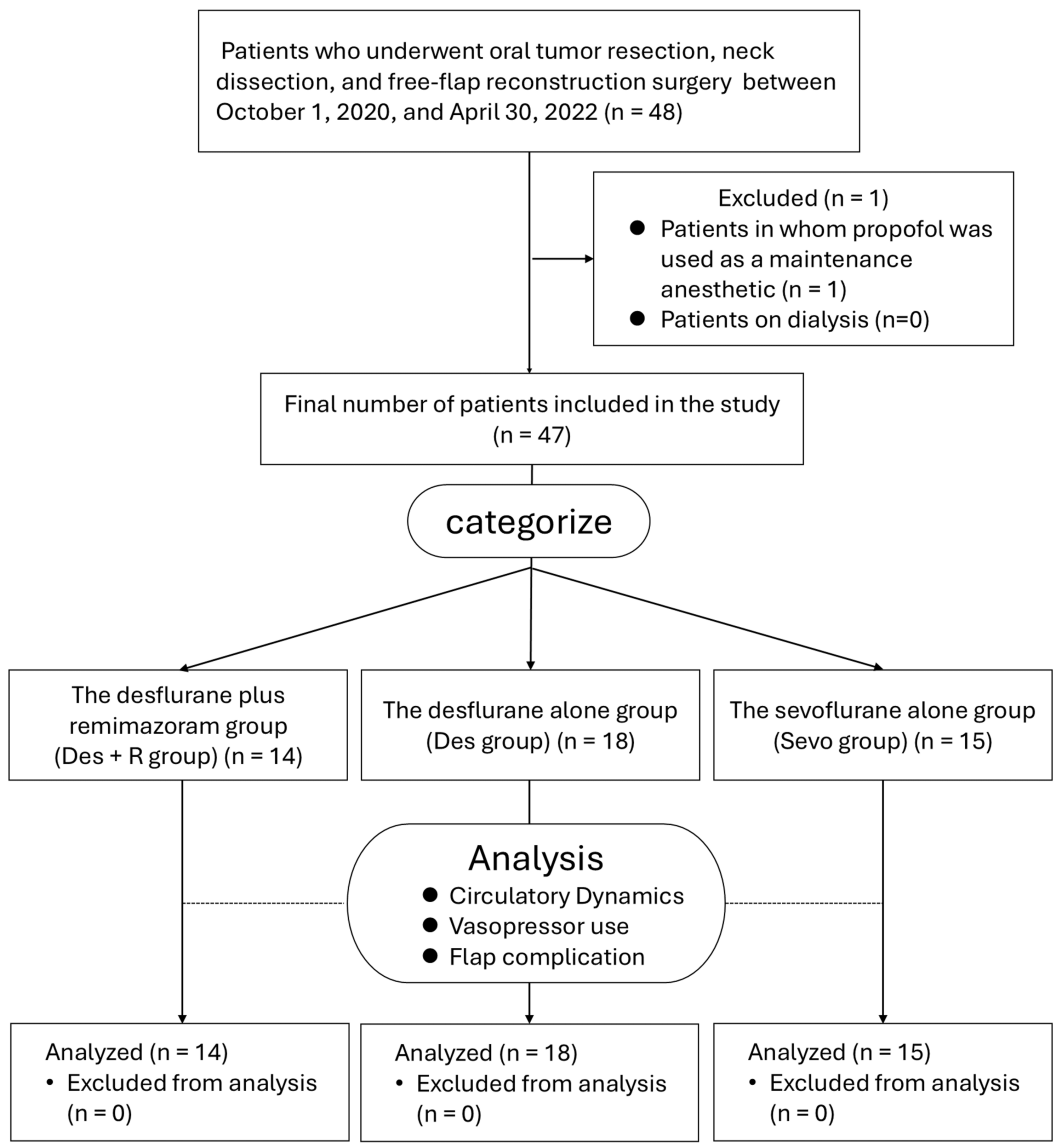
Flow diagram of the study.

In this study, data from 47 patients (Des+R group: n = 14; Des group: n = 18; Sevo group: n = 15) were analyzed, as shown in the flow diagram (Figure 1).

Patient background information is presented in Table 1.

**Table 1:** Demographic data of the study participants. Note: Continuous variables are presented as the median (interquartile range) and were compared using analysis of variance (ANOVA) or the Kruskal-Wallis test, followed by multiple comparison tests as appropriate. Categorical variables are expressed as the number (%) and were compared using Fisher’s exact test. *: There were overlapping comorbidities among patients. Des = desflurane; R = remimazolam besylate; Sevo = sevoflurane; MBP = pre-induction noninvasive mean arterial pressure; HR = pre-induction heart rate; Hb = pre-operative serum hemoglobin

	Des+R (n = 14)	Des (n = 18)	Sevo (n = 15)	Test statistic	P-value
Sex male, n (%)	8 (57)	9 (50)	11 (73)		0.39
Age, years	75.5 (70.5, 78.7)	72.5 (66.7, 80.5)	74.0 (62.5, 77.5)	F = 0.40	0.66
MAP (mmHg)	106.0 (89.0, 119.5)	103.0 (85.5, 115.2)	100.0 (93.5, 111.0)	F = 0.52	0.59
HR (beats/minute)	71.0 (64.5, 81.7)	75.5 (62.7, 84.0)	65.0 (63.5, 73.5)	F = 1.91	0.16
Hb (g/dL)	11.0 (10.0, 11.5)	11.4 (9.9, 12.3)	11.5 (10.9, 12.7)	F = 0.90	0.41
Medical history, n*					
Hypertension	7	11	8	-	-
Diabetes mellitus	0	7	1	-	-
Cardiac arrhythmia	2	2	3	-	-
Chronic heart failure	1	1	1	-	-
Valvular heart disease	0	2	0	-	-
Respiratory disease	0	2	1	-	-
Decreased renal function	3	1	2	-	-
Cerebral vascular disease/Dementia	3	1	2	-	-

The Des+R group included 8 (57%) males, the Des group included 9 (50%) males, and the Sevo group included 11 (73%) males (p = 0.39). The median age was 75.5 years (interquartile range (IQR) = 70.5, 78.7) for the Des+R group, 72.5 years (IQR = 66.7, 80.5) for the Des group, and 74.0 years (IQR: 62.5, 77.5) for the Sevo group (F = 0.85, p = 0.66). No significant differences were observed between the groups in terms of the sex ratio or age. There were no differences in noninvasive mean arterial pressure, heart rate, and serum hemoglobin among the groups in the “Pre-induction” period (F = 0.52, p = 0.59; F = 1.91, p = 0.4; F = 0.90, p = 0.41, respectively).

Anesthetic techniques

In the Des+R group, remimazolam was administered at 3-6 mg/kg/hour until loss of consciousness, then maintained at 1 mg/kg/hour. After endotracheal intubation, the remimazolam dose was gradually reduced to 0.1-0.2 mg/kg/hour before the start of surgery, while desflurane inhalation was initiated and maintained at 2-3%. Remimazolam administration was discontinued between 30 minutes before the end of surgery and the completion of the procedure. In the Des and Sevo group, propofol was used for induction, and after loss of consciousness, desflurane or sevoflurane was administered, with desflurane (4%) and sevoflurane (1.5%) as the basic dose. A BIS monitor was used to adjust the maintenance dose of inhaled anesthetics to maintain a BIS value of 40-60. In all groups, continuous infusions of remifentanil and single doses of fentanyl as needed were administered for analgesia.

Surgical and anesthetic management

Information on surgical and anesthetic management is presented in Table 2.

**Table 2:** Surgical and anesthetic data. Note: Continuous variables are presented as the median (interquartile range) and were compared using the Kruskal-Wallis test, followed by multiple comparison tests as appropriate. Des = desflurane; R = remimazolam besylate; Sevo = sevoflurane; etGAS = end-tidal anesthetic concentration; MAP = mean arterial pressure; HR = heart rate

	Des+R (n = 14)	Des (n = 18)	Sevo (n = 15)	Multiple comparison test	Test statistic	P-value
Anesthesia duration (minutes)	629 (579, 786)	697 (581, 871)	596 (545, 723)		Chi-squared = 1.5, df = 2	0.3
Surgical duration (minutes)	549 (505, 706)	611 (505, 774)	531 (469, 641)		Chi-squared = 1.5, df = 2	0.5
Fluid administration (mL/kg/hour)	6.6 (5.6, 7.7)	7.1 (5.2, 3.8)	6.0 (5.4, 7.4)		F = 0.54	0.5
Blood loss (mL)	593 (426, 874)	310 (208, 491)	336 (173, 495)		Chi-squared = 7.3, df = 2	0.03
				Des+R > Sevo	t = 2.40	0.04
Urine output (mL/kg/hour)	1.1 (0.8, 1.5)	0.8 (0.6, 2.3)	1.4 (0.9, 1.6)		Chi-squared = 0.51, df = 2	0.8
etGAS	2.1 (2.0, 2.7)	3.4 (3.1, 3.7)	1.3 (1.2, 1.3)			
BIS	47.3 (44.1, 49.3) (n = 14)	47.0 (42.6, 48.1) (n = 10)	54.5 (53.0, 55.0) (n = 4)			
“Pre-reconstruction” period						
MAP (mmHg)	77.1 (73.7, 82.5)	70.1 (68.9, 73.7)	70.7 (67.4, 75.0)		Chi-squared = 6.7, df = 2	0.04
				Des+R > Des	t = 2.35	0.04
HR (bpm)	72.8 (64.1, 84.9)	70.4 (63.6, 76.9)	64.9 (62.9, 69.5)			0.2
“Vascular anastomosis” period						
MAP (mmHg)	75.1 (71.0, 83.1)	68.0 (65.1, 71.6)	68.2 (63.3, 73.0)		Chi-squared = 8.2, df = 2	0.02
				Des+R > Des	t = 2.62	0.02
				Des+R > Sevo	t = 2.35	0.04
HR (beats/minute)	72.9 (68.2, 85.3)	74.0 (66.5, 80.7)	69.0 (66.2, 72.3)		F = 0.93	0.4

There were no significant differences among the groups in terms of anesthetic time, surgical time, fluid infusion volume, or urine output.

The median blood loss was 593 mL (IQR = 426, 874) for the Des+R group, 310 mL (IQR = 208, 491) for the Des group, and 336 mL (IQR = 173, 495) for the Sevo group (chi-squared = 7.3, df = 2, p = 0.03). Blood loss was significantly greater in the Des+R group than in the Sevo group (t = 2.40, p = 0.04).

There were no differences in heart rate among the groups in the “Pre-reconstruction” period and the “Vascular anastomosis” period (p = 0.2; F = 0.93, P = 0.4, respectively).

In the “Pre-reconstruction” period, the median mean arterial pressure was 77.1 mmHg (IQR = 73.7, 82.5) for the Des+R group, 70.1 mmHg (IQR = 68.9, 73.7) for the Des group, and 70.7 mmHg (IQR = 67.4, 75.0) for the Sevo group. The mean arterial pressure was greater in the Des+R group than in the Des group (t = 2.35, p = 0.04) before reconstruction. In the “Vascular anastomosis” period, the median mean arterial pressure was 75.1 mmHg (IQR = 71.0, 83.1) for the Des+R group, 68.0 mmHg (IQR = 65.1, 71.6) for the Des group, and 68.2 mmHg (IQR = 63.3, 73.0) for the Sevo group. The MAP was greater in the Des+R group than in the Des and Sevo groups (Des+R group vs. Des group: t = 2.63, p = 0.02; Des+R group vs. Sevo group: t = 2.34, p = 0.04) during vascular anastomosis.

Reoperations due to flap complications (thrombosis, hematoma, necrosis) were performed in two patients (three times) in the Des+R group, three patients (three times) in the Des group, and three patients (five times) in the Sevo group.

Relations with vasopressors

The use of vasopressors is presented in Table 3.

**Table 3:** Comparison of vasopressor use. Note: Continuous variables are presented as the median (interquartile range) and were compared using the Kruskal-Wallis test, followed by multiple comparison tests as appropriate. Categorical variables are expressed as the number (%) and were compared using Fisher’s exact test. Des = desflurane; R = remimazolam besylate; Sevo = sevoflurane

	Des+R (n = 14)	Des (n = 18)	Sevo (n = 15)	Multiple comparison test	Test statistic	P-value
Total dose of ephedrine (mg)	12.0 (8.0, 17.5)	26.0 (14.5, 46.0)	8.0 (6.0, 28.0)		Chi-squared = 8.4, df = 2	0.02
				Des > Des+R	t = 2.46	0.04
				Des > Sevo	t = 2.43	0.04
Total dose of phenylephrine (mg)	0.45 (0.23, 1.02)	0.90 (0.40, 2.19)	0.45 (0.07, 3.32)		Chi-squared = 0.57, df = 2	0.8
“Pre-reconstruction” period						
Continuous injection, n (%)	3 (21)	14 (77)	9 (60)			0.006
				Des > Des+R		0.005
Bolus injection, n						
Ephedrine	8	11	13			
Phenylephrine	12	12	9			
Noradrenaline	0	0	1			
Continuous injection, n						
Phenylephrine	3	6	5			
Noradrenaline	0	2	2			
Dopamine	1	7	4			
Dobutamine	0	0	1			
“Vascular anastomosis” period						
Continuous injection, n (%)	4 (28)	13 (72)	10 (66)			0.04
				Des > Des + R		0.03
Bolus injection, n						
Ephedrine	4	8	4			
Phenylephrine	4	10	5			
Noradrenaline	0	0	1			
Continuous injection, n						
Phenylephrine	3	4	1			
Noradrenaline	0	3	3			
Dopamine	1	6	6			
Dobutamine	0	0	1			

**Figure 2:**
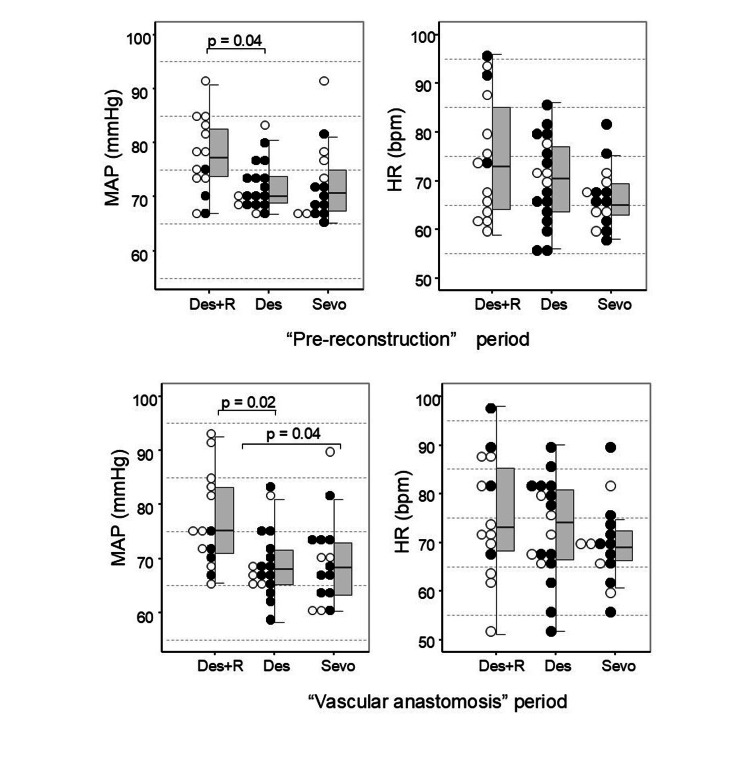
Comparison of anesthetic agents. The upper panel represents the pre-reconstruction phase, whereas the lower panel represents the vascular anastomosis phase. The mean arterial pressure (left graph) and heart rate (right graph) are plotted on the left side, with corresponding box plots displayed on the right. The horizontal line in the box plot represents the median. Filled circle: continuous vasopressor infusion. Circle: no continuous vasopressor infusion. The data were compared with those of the Steel-Dwass test. Des = desflurane; R = remimazolam besylate; Sevo = sevoflurane; MAP = mean arterial pressure; HR = heart rate

The results of the comparisons of the relationships between heart rate, mean arterial pressure, and the use of vasopressors with anesthetic agents are shown in Figure 2.

The rate of continuous vasopressor use in the “Pre-reconstruction” period was as follows: Des+R group: 21%; Des group: 77%; Sevo group: 60% (p = 0.006), and in the “Vascular anastomosis” period was: Des+R group: 28%; Des group: 72%; Sevo group: 66% (p = 0.04). The Des+R group had a significantly lower proportion of continuous vasopressor use in comparison to the Des and Sevo groups (“Pre-reconstruction” period: p = 0.005; “Vascular anastomosis” period: p = 0.03).

**Figure 3:**
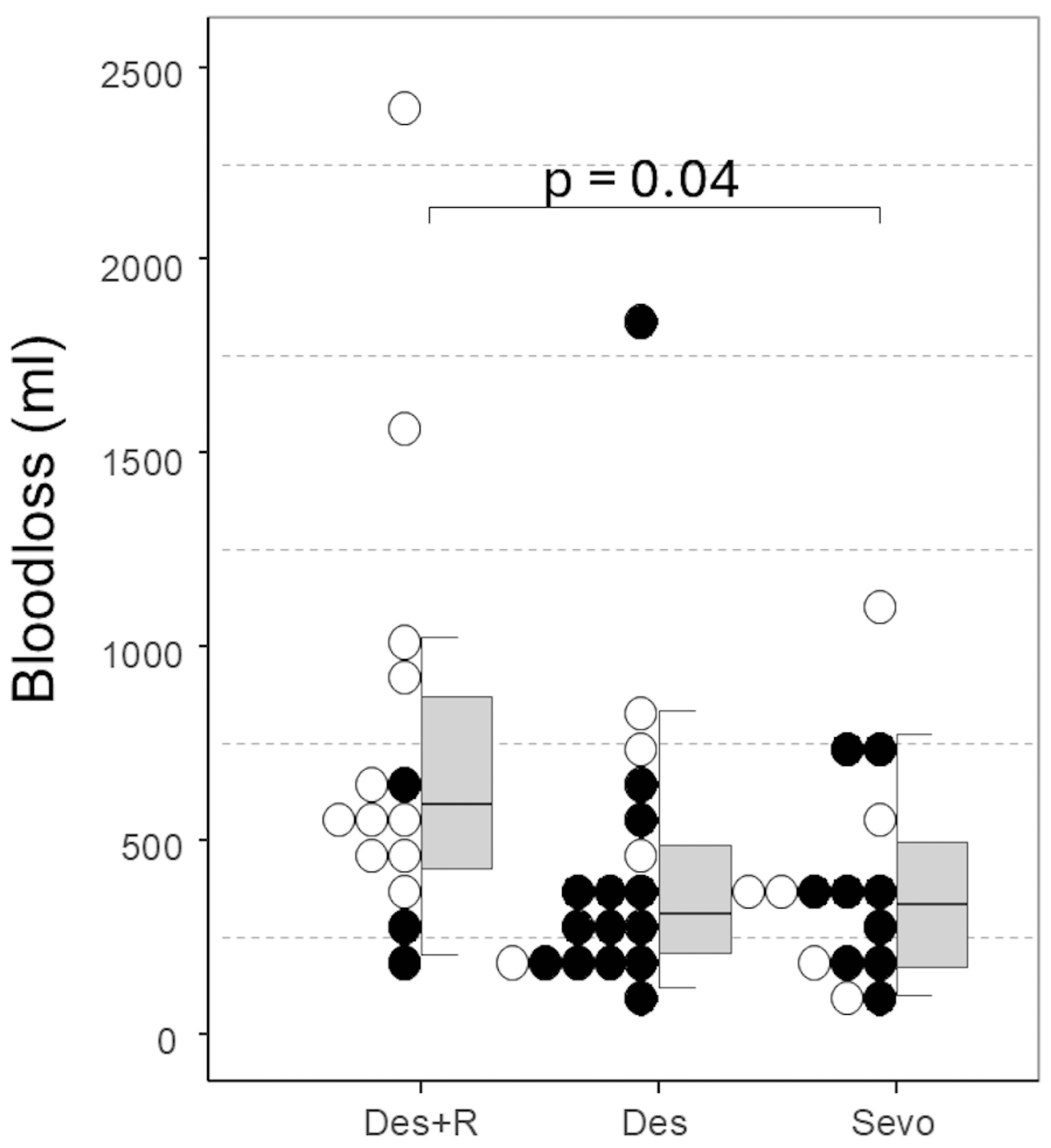
Comparison of blood loss by anesthetic agent. Blood loss is plotted on the left side, with the corresponding box plot displayed on the right. The horizontal line in the box plot represents the median. Filled circle: continuous vasopressor infusion. Circle: no continuous vasopressor infusion. The data were compared with those of the Steel-Dwass test. Des = desflurane; R = remimazolam besylate; Sevo = sevoflurane

The amount of ephedrine used (Des+R group: 12.0 mg (IQR = 8.0, 17.5); Des group: 26.0 mg (IQR: 14.5, 46.0); Sevo group: 8.0 mg (IQR = 6.0, 28.0); chi-squared = 8.4, df = 2) was significantly greater in the Des group than in the Des+R and Sevo groups (Des+R group vs. Des group: t = 2.46, p = 0.04; Des group vs. Sevo group: t = 2.43, p = 0.04), whereas no significant differences in the amount of phenylephrine used were detected among the groups (chi-squared = 0.57, df = 2, p = 0.8). The results of a comparison of the relationship between blood loss and the presence or absence of continuous vasopressor administration by anesthetic agent are shown in Figure 3, indicating a tendency toward lower blood loss when continuous vasopressor administration was performed.

## Discussion

Previous reports have examined the hemodynamic profile and vasopressor use during total intravenous anesthesia with remimazolam [9,10]; however, no reports have compared the effects of remimazolam combined with inhalation anesthetics on hemodynamics with those of conventional anesthesia methods. In this study, intraoperative blood pressure was maintained at a high level, and vasopressor use was lower when remimazolam was used in combination, although intraoperative blood loss was greater.

Intravenous anesthetics and inhalation anesthetics have distinct mechanisms of action, and their combination may offer the advantages of both agents, potentially providing safer and more efficient anesthesia. Intravenous anesthetics are generally used for rapid induction of anesthesia, whereas inhalation anesthetics are used for stable maintenance of anesthesia. However, when used in combination for anesthesia maintenance, intravenous anesthetics and inhalation anesthetics may potentiate each other’s effects, requiring caution. Recently, remimazolam, a short-acting anesthetic with minimal circulatory depression, has been marketed and is considered suitable for combination with inhalation anesthetics. While the combination of remimazolam and analgesics has been shown to suppress intraoperative hypotension, the effects of combining remimazolam with inhalation anesthetics on hemodynamics remain poorly understood. The purpose of this study was to evaluate the effects of remimazolam on circulatory management during free-flap reconstruction surgery and to verify its usefulness in anesthetic management. The results of this study also revealed that blood pressure was maintained at a high level in the remimazolam group, suggesting that it may be an appropriate anesthetic method for flap creation surgery.

In free-flap reconstruction for oral cancer, the management goals differ before and after reconstruction. During neck dissection and tumor resection, adequate anesthesia depth and analgesia are necessary to suppress blood pressure elevation. On the other hand, during reconstruction involving vascular anastomosis, blood pressure must be maintained at a higher level to preserve flap perfusion. Therefore, this study analyzed data separately for the pre-reconstruction and vascular anastomosis periods.

The mean arterial pressure before reconstruction (neck dissection and tumor resection) was 77.1 mmHg in the Des+R group, 70.1 mmHg in the Des group, and 70.7 mmHg in the Sevo group. Intraoperative hypotension is associated with an increased incidence of postoperative myocardial injury and acute kidney injury, and the avoidance of hypotension with a mean arterial pressure below 60-70 mmHg is recommended [1]. Inhalation anesthetics alone require the administration of vasopressors to avoid intraoperative hypotension, but in the remimazolam combination group, many patients did not require vasopressors. Inhalation anesthetics produce loss of consciousness and circulatory depression at different concentrations. Loss of consciousness is generally achieved at approximately 0.5 MAC, reflecting central nervous system depression, whereas circulatory depression, mediated by myocardial depression, vasodilation, and attenuation of autonomic reflexes, becomes prominent at 1 MAC or higher. These thresholds are reduced in elderly patients and further lowered with the concomitant administration of opioids such as remifentanil or fentanyl [11]. In the present study, by combining remimazolam and maintaining the inhalation anesthetic concentration below the level at which circulatory depression occurs, blood pressure could be maintained without the need for vasopressor support.

On the other hand, the Des+R group showed significantly greater blood loss. This may be due to the effects of increased blood pressure or the extent of vasoconstriction. However, although the mean arterial pressure was significantly greater in the Des+R group, the difference was not sufficient to account for the difference in blood loss. In this study, a trend toward lower blood loss was observed in the group receiving continuous vasopressor administration, suggesting that vasopressor-induced vasoconstriction may have contributed to reduced blood loss. In other words, at equivalent blood pressure levels, the use of vasopressors may be effective in suppressing blood loss. However, this was a retrospective study, and the use of vasopressors was left to the discretion of the anesthesiologists. Therefore, there was no uniformity in the type of vasopressors or the method of their administration, making it difficult to clearly evaluate the effect of vasopressors on blood loss. In the future, a prospective study under standardized criteria is necessary to further investigate the effects of vasopressor type and administration method on blood loss.

The mean arterial pressure during vascular anastomosis in this study was 75.1 mmHg in the Des+R group, 68.0 mmHg in the Des group, and 69.0 mmHg in the Sevo group. In free-flap reconstruction, blood pressure and fluid management to maintain flap perfusion are important. It has been reported that maintaining a mean arterial pressure of 70-80 mmHg during vascular anastomosis helps maintain perfusion pressure [12,13]. During vascular anastomosis, blood pressure often decreases due to surgical trauma, necessitating the use of vasopressors to maintain circulation. In this study, blood pressure was maintained without continuous administration of vasopressors in the Des+R group, whereas continuous administration of vasopressors was required to maintain blood pressure in the Des and Sevo groups (Figure 1). Previous reports indicate that the vasopressor usage rate during free-flap reconstruction surgery ranges from 53% to 85% [14], which is consistent with the rates of 72% in the Des group and 66% in the Sevo group. On the other hand, the rate in the Des+R group was 28%, which was lower than that reported in previous studies. Previous studies have discussed the effects of vasopressor use on flap blood flow [3,12,15]. It has been suggested that vasopressor-induced peripheral vasoconstriction may worsen flap blood flow; however, many studies have shown no association between free tissue failure and intraoperative vasopressor use [3,16]. However, as single doses can cause rapid vasoconstriction, the continuous administration of low-dose vasopressors is recommended [15].

This study demonstrated that the combination of remimazolam with inhaled anesthetics minimizes the use of vasopressors while maintaining hemodynamic stability. The use of vasopressors is of concern because of their vasoconstrictive effects on flap creation [17,18]. The combination of remimazolam and inhaled anesthetics is considered useful from the perspective of maintaining flap blood flow. In the future, optimal management of flap perfusion can be provided by adjusting anesthetics, vasopressors, and fluid volume based on indicators such as cardiac output and vascular resistance in combination with advanced hemodynamic monitoring. From the perspective of blood loss, adjusting the maintenance dose of anesthetics before and after reconstruction to appropriately manage blood pressure should also be considered when remimazolam is used in combination with desflurane.

Because the choice of anesthetic regimen was at the discretion of the attending anesthesiologists, the potential for selection bias cannot be excluded. It is possible that patients with greater comorbidity, anticipated bleeding tendency, or other intraoperative risks were preferentially assigned to one regimen over another. Therefore, the observed differences in hemodynamic stability or blood loss may partly reflect baseline clinical characteristics rather than the pharmacologic properties of the anesthetic agents themselves. Although we compared baseline variables among groups, unmeasured confounders may still have influenced the results. Future prospective studies or analyses using propensity score adjustment will be necessary to minimize such bias and clarify whether the observed associations are causal.

Limitations

The present study was associated with several limitations, including the small number of cases (n = 47); the single-center setting; the lack of uniformity in the types and administration methods of vasopressors, which prevented accurate evaluation of their effects; the lack of evaluation of the effects of the combination of sevoflurane and remimazolam; and the inadequate evaluation of sedation depth. Because this was a retrospective observational study, the sample size was determined by the available cases, and no a priori calculation was performed. We ensured that each group included at least 10 patients to allow for multiple comparisons. The present findings should be interpreted with caution, but they may provide useful information for future prospective trials in which formal sample size estimation will be conducted. Although the BIS was used to evaluate sedation, the reference electroencephalography for BIS measurement did not include data on remimazolam or desflurane, limiting the reliability of the BIS. Additionally, in head and neck surgery, noise from surgical maneuvers may interfere with BIS measurements, making accurate assessment challenging. The level of sedation when remimazolam was used was confirmed to be appropriate based on the BIS waveform and was comprehensively evaluated with reference to other vital signs.

## Conclusions

In free-flap reconstruction surgery for oral cancer, the combination of remimazolam and desflurane anesthesia suggested the potential to avoid intraoperative hypotension without relying on vasopressors. To clarify whether this combination is suitable as an anesthetic technique for free-flap reconstruction in oral cancer, appropriately designed prospective observational studies are needed, including strict protocols for vasopressor administration and postoperative evaluation of flap-related complications.
